# Comparison of pre-analytical characteristics for molecular and serological diagnostics of COVID-19

**DOI:** 10.3205/dgkh000374

**Published:** 2021-01-19

**Authors:** Ralph Wendt, Olaf Nickel, Sven Kalbitz, Jasmin Fertey, Sebastian Ulbert, Johannes Wolf, Christoph Lübbert, Stephan Borte

**Affiliations:** 1 Department of Infectious Diseases/Tropical Medicine, Nephrology and Rheumatology, Hospital St. Georg, Leipzig, Germany; 2 Department of Laboratory Medicine, Hospital St. Georg, Leipzig, Germany; 3 Fraunhofer Institute for Cell Therapy and Immunology (IZI), Leipzig, Germany; 4 ImmunoDeficiencyCenter Leipzig (IDCL) at Hospital St. Georg Leipzig, Jeffrey Modell Diagnostic and Research Center for Primary Immunodeficiency Diseases, Leipzig, Germany; 5 Division of Infectious Diseases and Tropical Medicine, Department of Medicine II, Leipzig University Hospital, Leipzig, Germany; 6 Interdisciplinary Center for Infectious Diseases, Leipzig University Hospital, Leipzig, Germany; 7 Department of Laboratory Medicine, Division of Clinical Immunology, Karolinska Institute, Stockholm, Sweden

**Keywords:** SARS-CoV-2, PCR, swabs, pharyngeal lavage, COVID-19

## Abstract

**Background:** The diagnosis of SARS-CoV-2 infection relies on RT-PCR from nasopharyngeal swabs. The pre-analytical value of different methods of material harvesting for SARS-CoV-2 are unknown.

**Methods:** We conducted a comprehensive investigation of the pre-analytical performance for different pharyngeal sampling procedures in hospitalized patients with confirmed SARS-CoV-2 infection. In addition to swabs taken simultaneously from different locations, saliva and pharyngeal lavages were also analyzed using RT-PCR.

**Results:** In 10 COVID-19 patients, standard nasopharyngeal swabs detected 8 out of 10 positive patients, whereas swabs taken from the palatoglossal arch resulted in 9 correct-positive results. Brushing the posterior pharynx wall with swabs resulted in detection of 9 out of 10 positive patients with no difference using either dry swabs or liquid Amies medium. A strong correlation between Ct values of both swab materials was observed. Pharyngeal lavages yielded 6 out of 10 positive results in concordance with 85% of nasopharyngeal swabs in late-stage COVID-19 patients. Investigating 23 patients with early SARS-CoV-2 infection, pharyngeal lavages showed a concordance rate of 100% compared to nasopharyngeal swabs.

**Conclusions:** The diagnostic performance of swabs taken from the palatoglossal arch in detecting SARS-CoV-2 infection is similar to that of specimens taken from the nasopharyngeal region. However, the former sampling method is associated with less discomfort and much easier to perform. Pharyngeal lavages may replace swabs for mass screening in early stages of SARS-CoV-2 infection. The predictive values are comparable, and the procedure is performed without exposing healthcare workers to transmission risks.

## Zusammenfassung

**Hintergrund:** Die Diagnose der SARS-CoV-2-Infektion basiert auf einer RT-PCR aus Nasen-Rachen-Abstrichen. Der präanalytische Wert der verschiedenen Methoden der Materialentnahme für die Diagnose einer SARS-CoV-2 Infektion ist unbekannt.

**Methoden:** Wir führten eine umfassende Untersuchung der präanalytischen Wertigkeit verschiedener pharyngealer Probeentnahmeverfahren bei hospitalisierten Patienten mit bestätigter SARS-CoV-2-Infektion durch. Neben Abstrichen mit verschiedenen Materialien, die gleichzeitig an verschiedenen Orten entnommen wurden, wurden auch Speichel- und Rachenspülungen mittels RT-PCR analysiert.

**Ergebnisse:** Von 10 COVID-19 Patienten wurden mittels standardisierten Nasen-Rachen-Abstrichen 8 von 10 Patienten korrekt erkannt, während Abstriche vom vorderen Rachenring zu 9 korrekt-positiven Ergebnissen führten. Abstriche der hinteren Rachenwand führten bei 9 von 10 Patienten zum korrekten SARS-CoV-2 Nachweis ohne Unterschiede bei Verwendung von trockenen Abstrich-Materialien oder Abstrichtupfern mit flüssigem Amies-Medium. Es wurde eine starke Korrelation zwischen den Ct-Werten bei Verwendung der beiden Abstrichmaterialien beobachtet. Bei COVID-19-Patienten im Spätstadium wurden mittels Rachenspülungen 6 von 10 Patienten korrekt zugeordnet, während dies bei 85% der Nasen-Rachenabstriche der Fall war. Bei der Untersuchung von 23 Patienten mit einer frühen SARS-CoV-2-Infektion zeigten die Rachenspülungen im Vergleich zu den Nasen-Rachen-Abstrichen eine Konkordanzrate von 100%.

**Schlussfolgerung:** Abstriche, die vom vorderen Rachenring entnommen werden, weisen bei Patienten mit SARS-CoV-2-Infektion eine ähnliche diagnostische Sicherheit auf wie Proben aus dem Nasen-Rachenraum-Bereich. Diese Probenentnahmemethode ist jedoch deutlich weniger unangenehm und einfacher durchführbar. Rachenspülungen können in frühen Stadien einer SARS-CoV-2-Infektion Nasenrachenabstriche für das Massenscreening ersetzen. Die prädiktiven Werte sind vergleichbar und das Gesundheitspersonal wird nicht unnötig einem Übertragungsrisiko ausgesetzt.

## Introduction

To identify patients and carriers with SARS-CoV-2 infection, widespread diagnostic testing is of paramount importance. Among other factors, the diagnostic performance of applicable tests depends on the clinical stage of disease and the quality of harvested material. Currently, the most commonly applied method in SARS-CoV-2 testing is using swab material harvested through the nasopharyngeal route or deep pharyngeal brushing. Both procedures must be performed by sufficiently trained healthcare workers (HCWs) and may result in sampling discomfort for the patients and potential exposition of HCW to contagious droplets or even aerosols. The pre-analytical value of different sampling methods and locations have yet to be evaluated. 

## Methods

### Diagnostic protocol

**Figure 1:**
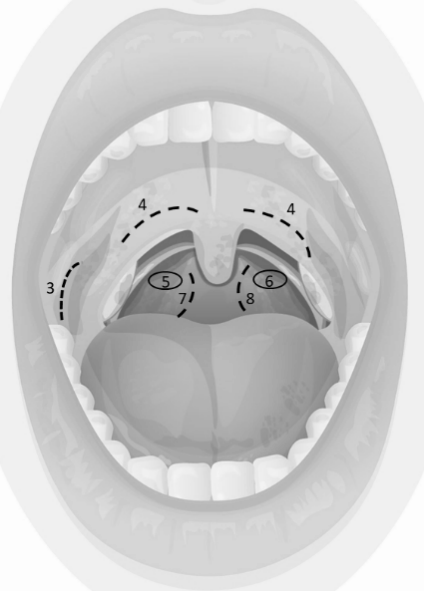
locations of pharyngeal material harvest by swab touching (circles) or brushing (dotted lines)

Copan eSwabs and dry swabs (both Copan S.p.A., Brescia, Italy) were used to simultaneously brush mucosal cells at different locations of the pharynx (Figure 1 (Fig. 1)) in hospitalized COVID-19 patients. Additionally, pharyngeal lavage was performed at baseline (t0), after 5 minutes (t5) and after ingestion of a full meal (tm). 

The pharyngeal lavage was harvested by providing the patient with a receptacle containing 10 mL of 0.9% saline solution. The patients were instructed to gargle intensely with the solution for 10 seconds and then spit the recovered liquid into the same receptacle, immediately followed by closure and transport to the laboratory. Pharyngeal lavage samples were analyzed directly after arrival or stored at 4°C and re-analyzed after 48 h (stability testing).

All procedures and material extractions were performed by a single, well-trained, experienced investigator. Within a few minutes, the following procedures were accomplished:

1. nasopharyngeal eSwab left;
2. nasopharyngeal dry swab right;
3. buccal eSwab;
4. eSwab of the palatoglossal arch,
5. eSwab of the left posterior pharyngeal wall (just touching, no brushing);
6. dry swab of the right posterior pharyngeal wall (just touching, no brushing);
7. eSwab of the left posterior pharyngeal wall left (brushing);
8. dry swab of the right posterior pharyngeal wall (brushing),
9. Saliva (patient spits into a receptacle);
10. pharyngeal lavage;
11. pharyngeal lavage 5 minutes after 10);
12. pharyngeal lavage after eating and drinking.

Additionally, we performed 23 paired-tests of nasopharygeal eSwabs and pharyngeal lavage in known COVID-19 patients with early-stage SARS-CoV-2 infection as defined by a previous RT-PCR with a Ct value <30 within the past 24 hours.

Furthermore, eSwab samples from 8 randomly chosen hospitalized COVID-19 patients were resuspended in DMEM medium supplemented with 1% penicillin/streptomycin and immediately subjected to VERO cell transduction assays within 4 hours. 

### Molecular and serological analyses

To detect SARS-CoV-2 infection, either Copan Liquid Amies eSwabs or conventional dry swabs as well as pharyngeal lavage were subjected to cellular lysis and RNA extraction, followed by RT-PCR. RNA extraction was performed on the CyBio Felix 96-well plate system (AJ Roboscreen, Leipzig, Germany), and real-time RT-PCR was conducted using LightCycler Multiplex RNA Virus Master Mix on a Lightcycler 480 RT system (both Roche, Mannheim, Germany) or a ViiA7 system (Applied Biosystems, Foster City, CA, USA).

For all analyses, a Sarbecovirus-specific LightMix Modular SARS-CoV (COVID-19) E gene assay was used (TIB Molbiol, Berlin, Germany) [1]. The E gene assay was validated with an E gene control (*in vitro* transcribed E gene RNA), as well as SARS-CoV-1 strain Frankfurt 1 RNA control, and a SARS-CoV-2 strain RNA control (European Virus Archive GLOBAL). EAV extraction control (TIB Molbiol, Berlin, Germany) was used as internal PCR control. 

SARS-CoV-2 nucleocapsid antibody (IgA, IgG and IgM) detection was performed using a CE-IVD certified ELISA (Virotech, Rüsselsheim, Germany) with sera taken at least 7 days after onset of symptoms. SARS-CoV-2 nucleocapsid antibody-negative samples were re-tested with an S1-protein-specific CE-IVD certified ELISA (Euroimmun Lübeck, Germany). Automated processing and signal detection were processed on a DSX platform (DYNEX Technologies, Chantilly, USA). 

### Vero cell viral transduction assay

Vero E6 cells (obtained from DSMZ German Collection of Microorganisms and Cell Cultures, Braunschweig, Germany) were cultured in DMEM medium (ThermoFisher Scientific, Germany), supplemented with 10% heat-inactivated FBS (Gibco, Germany) and 1% penicillin/streptomycin (Gibco, Germany). For virus cultivation, 2x10^5^ Vero E6 cells per well were seeded in 6-well plates (Greiner, Germany) the day before infection. Virus propagation was performed in a BSL-3 lab. On the day of infection, the medium was removed and the cell layer was washed with 1xPBS before inoculation with 500-µl swab sample per well. Inoculation was performed in duplicates. Fifty microliters of a SARS-CoV-2 isolate (BetaCoV/Germany/BavPat1/2020 p.1) with 1x10^5^ pfu/ml was inoculated in parallel and served as positive control; non-infected cells served as negative control. After 1 h at 37°C, 2 ml DMEM +2% FBS and 1% penicillin/streptomycin were added to each well. The medium was exchanged after 20 h. After 3 days, 1 ml of cell culture supernatant was passaged on fresh 6-well plates containing 2x10^5^ Vero E6 cells per well and mixed with 1 ml DMEM +2% FBS and 1% penicillin/streptomycin. The samples were cultivated for 13 days with two subsequent passages of the cell culture supernatant. Development of a cytopathic effect was monitored by microscope before passaging. In three samples, fungal contamination was visible and 2.5 µg/ml Fungizone was added to the medium for all further steps. After removing the cell culture supernatant, cells were fixed with 70% ethanol for 30 minutes and stained with crystal violet. Stained plates were documented using a luminous plate and a gel documentation system (Intas GDS, INTAS Science Imaging Instruments, Göttingen, Germany).

### Statistical analysis

Only descriptive statistics were applied. Numerical variables were summarized as means with standard deviations, and categorical variables were given as frequencies or proportions. SPSS version 24.0 (IBM, Armonk, NY, USA) was used for statistical calculations. Correlation analyses were performed using Pearson’s correlation coefficient. For non-parametric tests, the Mann-Whitney U-test was applied.

### Ethical approval

This study was performed in accordance with the ethical guidelines of the 1964 Declaration of Helsinki and its later amendments, and was approved by the local institutional review board (Ethics Committee of the Saxonian Board of Physicians, EK-BR-48/20-1).

## Results

Three of 10 patients had severe courses of COVID-19, and 7 patients had moderate disease, according to clinical severity categories that correspond to the World Health Organization’s (WHO’s) classification. The mean age was 71 ±17 years. 60% were men, 40% women, all had at least one of the following comorbidities: arterial hypertension, diabetes mellitus, chronic obstructive pulmonary disease (COPD), atherosclerotic vascular disease. The mean time from first onset of symptoms to hospitalization was 10 ±5 days. The mean cycle threshold (Ct) value of positive SARS-CoV-2 PCRs in this investigation was 27.5 ±7.1. For all 10 patients, SARS-CoV-2-specific antibody production was observed ( see Supplementary Table 2 ). 

### Swab location and method of material extraction

**Figure 2:**
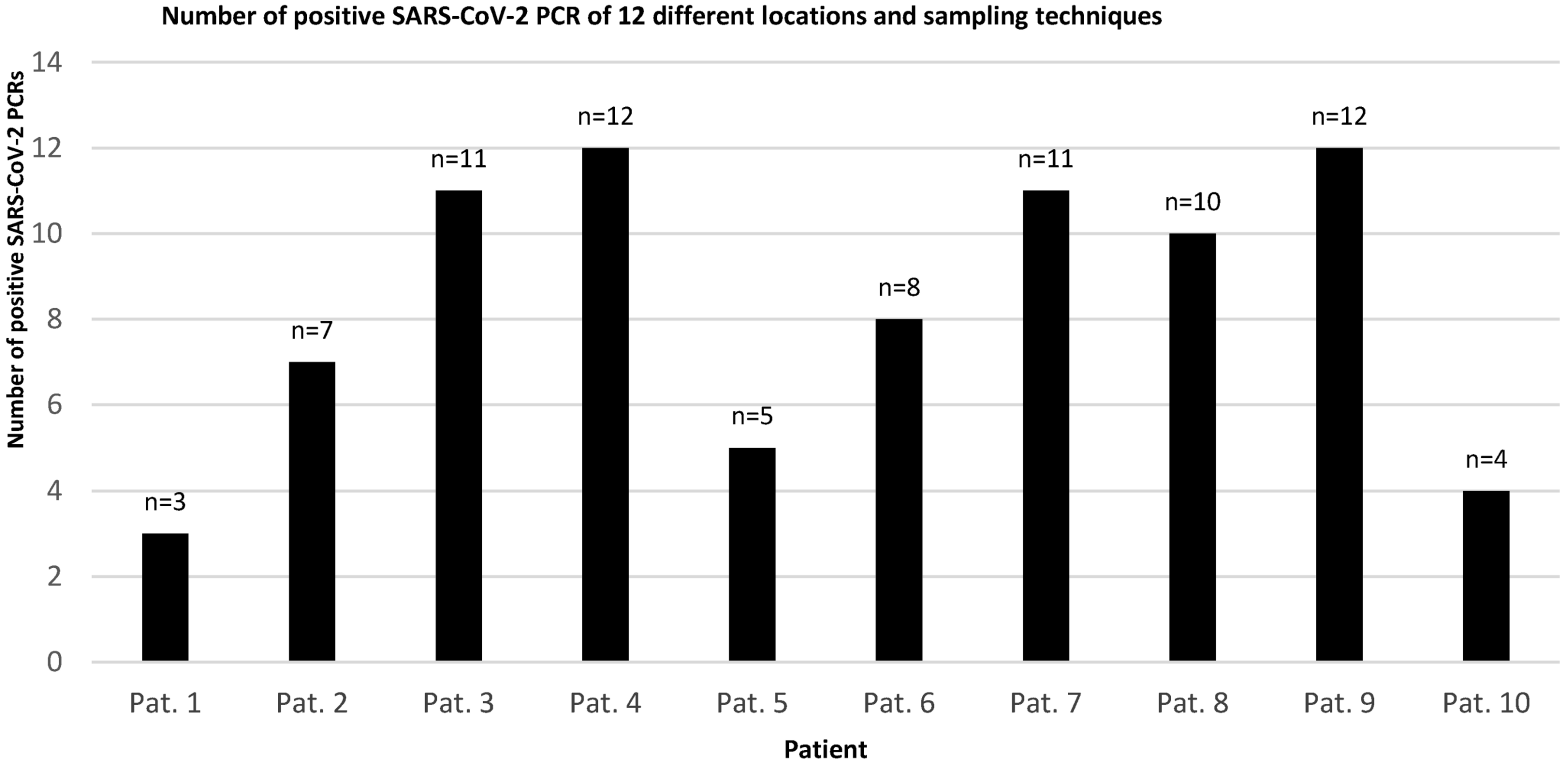
Number of positive detections of SARS-CoV-2 in PCR from material harvested at different nasopharyngeal locations in 10 known SARS-CoV-2-positive patients

**Figure 3:**
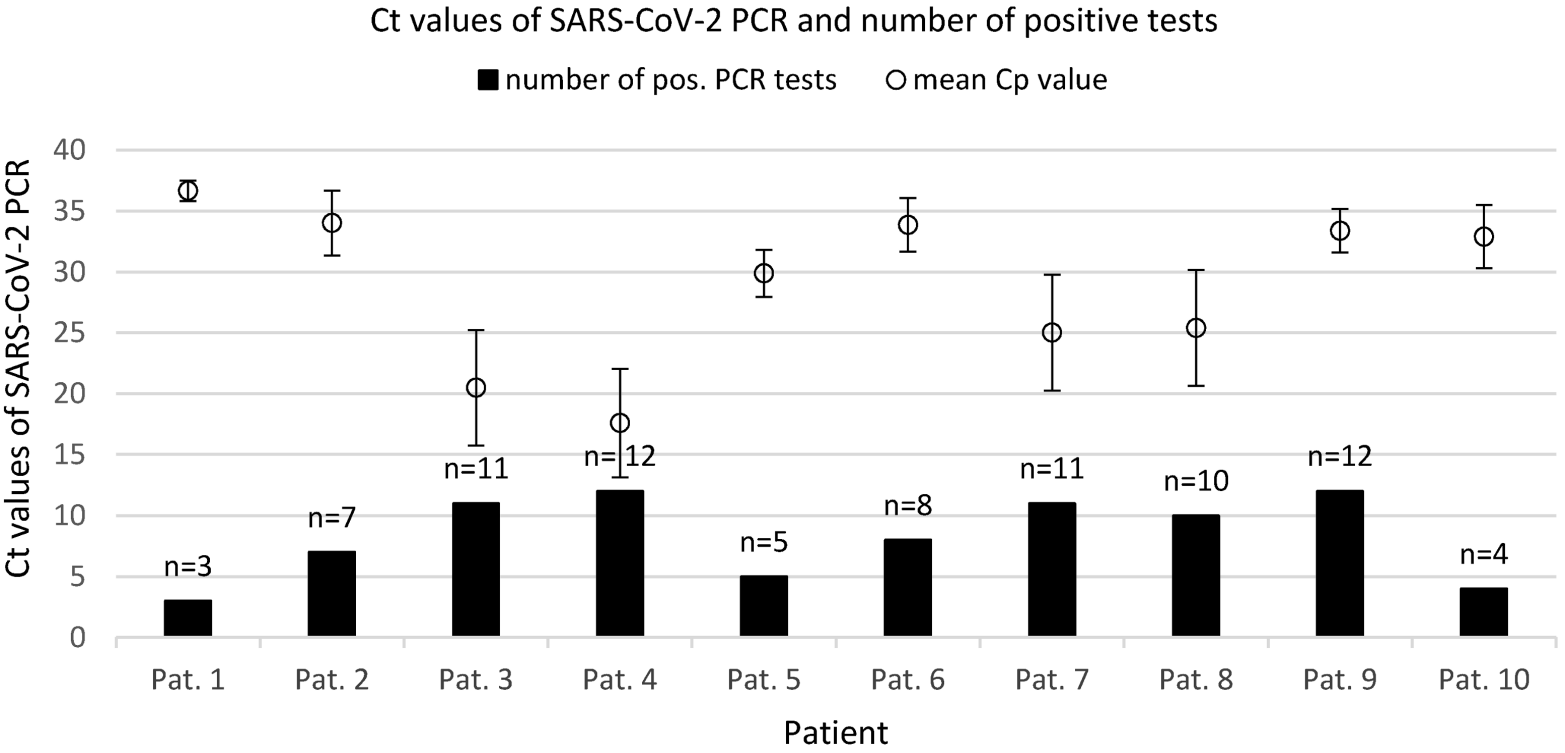
Mean Ct values per tested patient of positive PCR tests and number of positive SARS-CV-2 PCR from the 12 different material sampling techniques and locations in each of the known SARS-CoV-2-positive patients

As depicted in Figure 2 (Fig. 2) and Figure 3 (Fig. 3), lower Ct values of SARS-CoV-2 PCR and thus higher assumed viral concentrations were associated with higher detection rates among all material extraction locations and techniques. In patients #3, #4, #7 and #8, the Ct values of nasopharyngeal swabs (the reference method) were below 20 (15,8, 13,4, 18,1 and 15,6 respectively), and the average SARS-CoV-2 positivity frequency using different methods and locations of material extraction was 11 out of 12 (91.7%). Patient 9 was exceptional in terms of a maximum Ct value of 34.1 on day 21 of the disease and 12 out of 12 positive test results applying all harvesting methods. 

**Figure 4:**
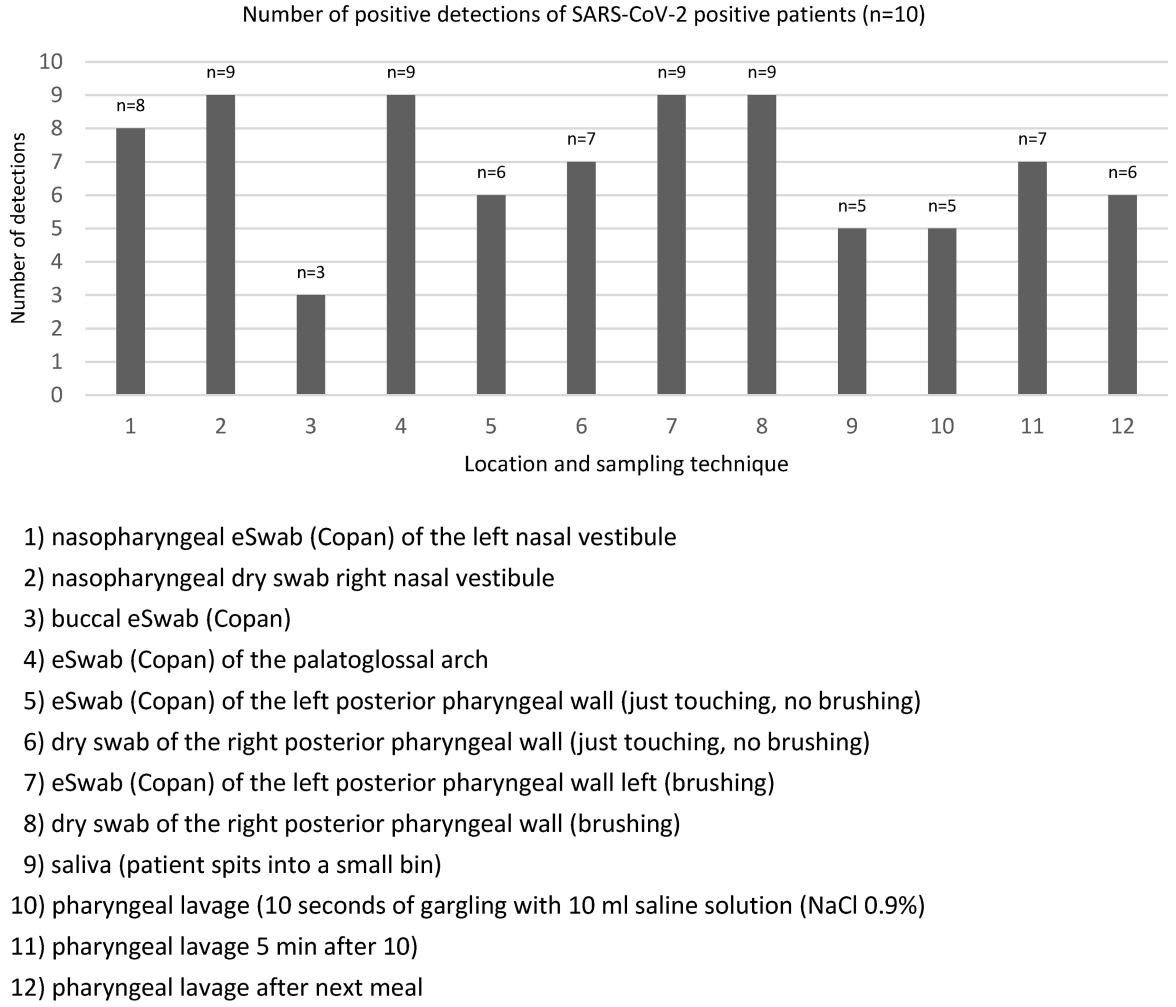
Number of positive detections of SARS-CoV-2 in PCR from 10 known SARS-CoV-2-positive patients using different material harvesting techniques and locations

**Figure 5:**
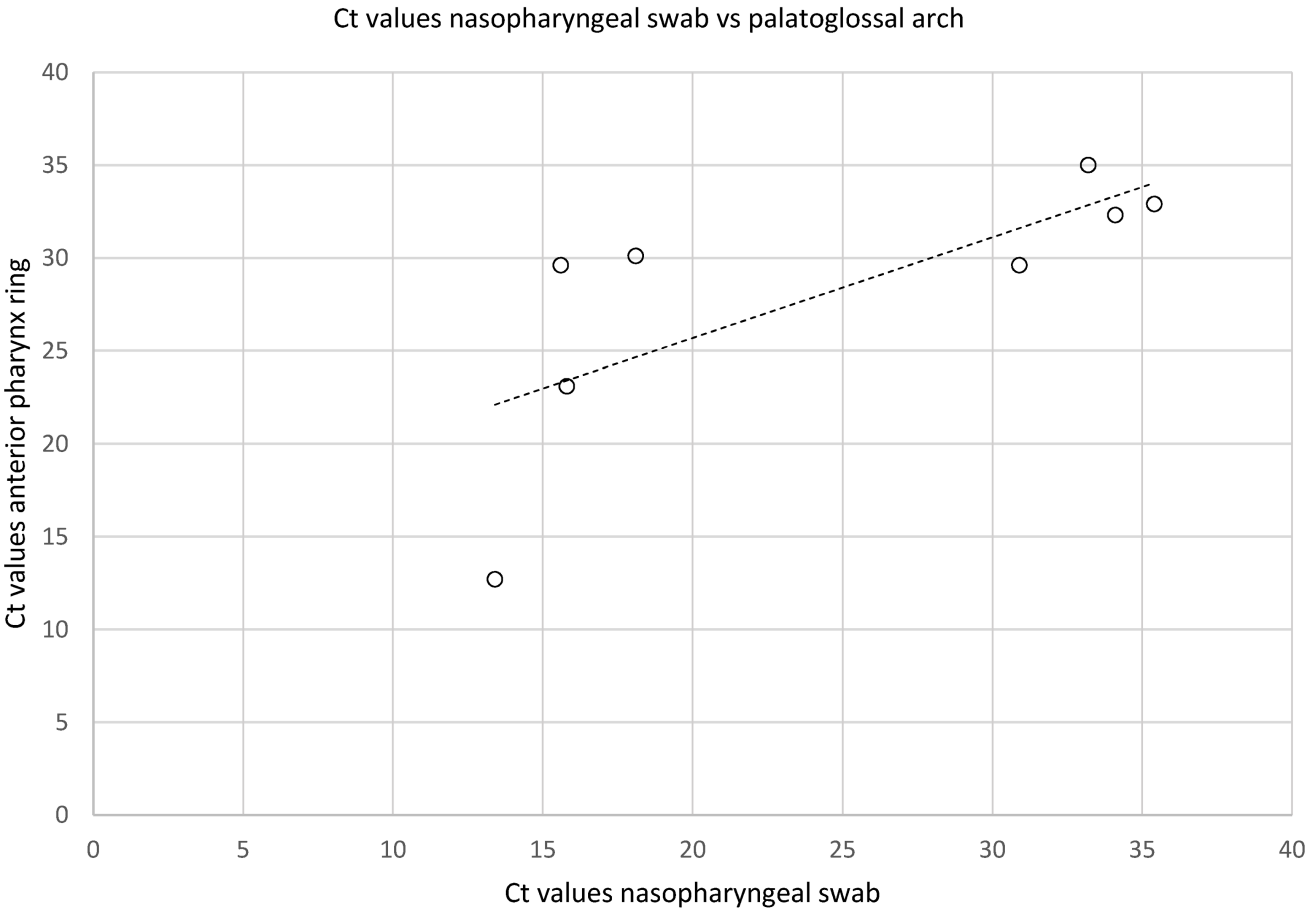
Correlation between Ct values of SARS-CoV-2 PCR from swabs of the anterior pharynx ring and nasophyngeal swabs in known SARS-CoV-2 positive patients, R²=0.53

The highest SARS-CoV-2 detection rates were found taking swabs from the palatoglossal arch, from the nasopharynx, and by brushing the posterior pharynx wall, irrespective of the swab system used (Figure 4 (Fig. 4)). The lowest detection rate was noted in swabs taken from the buccal mucosa. There was a strong correlation between Ct values of nasopharyngeal swab and material extracted from the palatoglossal arch (R^2^=0.53) (Figure 5 (Fig. 5)).

Compared to anatomical regions with a high detection rate, pharyngeal lavage had a lower detection rate in the whole cohort (6 out of 10 positive detections). In contrast, the SARS-CoV-2 detection rate in patients with nasopharyngeal-swab Ct values <30 using pharyngeal lavage was 100%. Repeated pharyngeal lavage shortly after the first harvest and after eating a meal did not significantly influence the detection rate.

### Dry swabs versus eSwabs (Copan) with Amies transport medium

**Figure 6:**
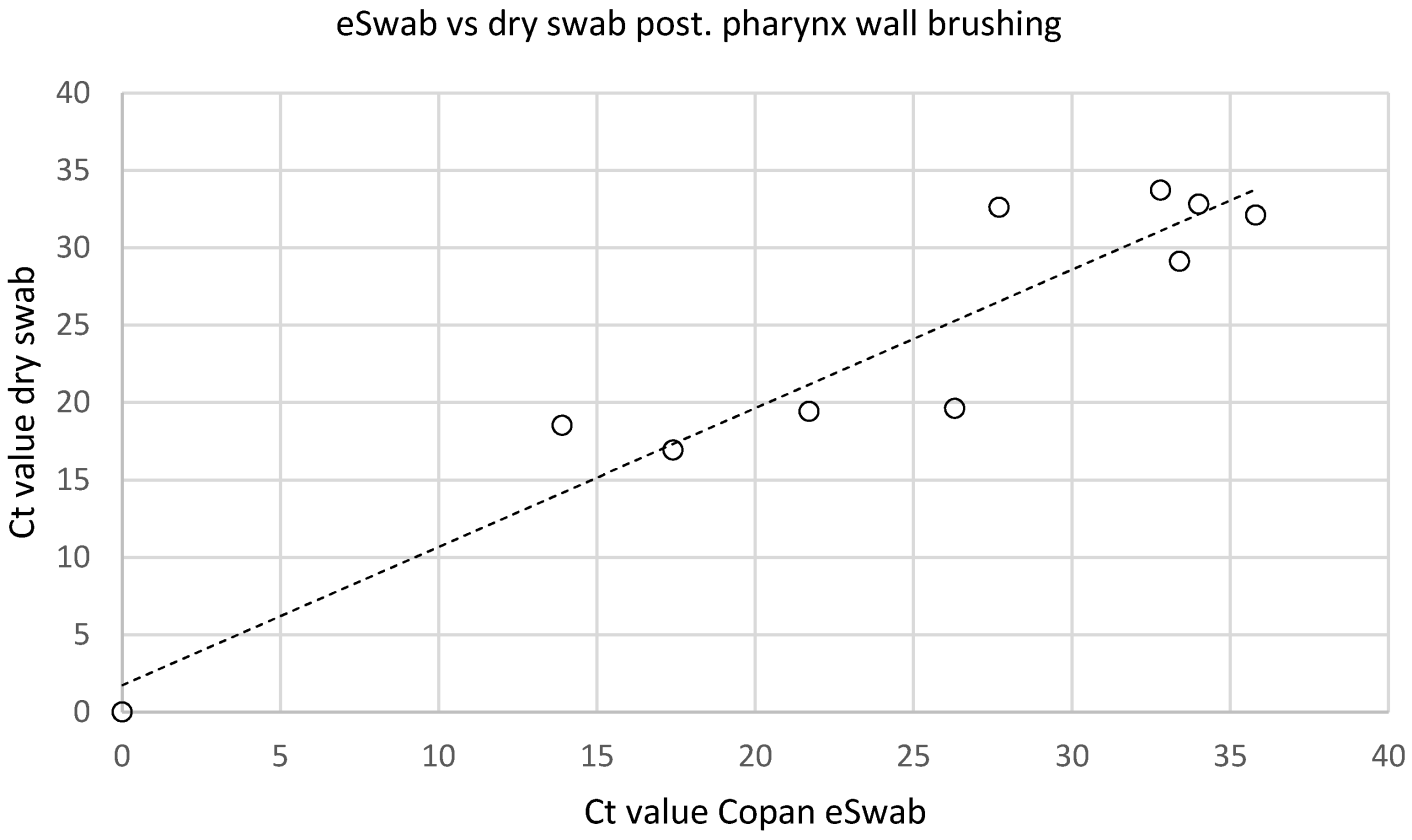
Correlation of dry versus copan eSwabs from the same location (posterior pharynx wall, brushed left and right), R²=0.89

There was no difference between the two types of swabs used. In 10 patients, the two different swab systems (eSwab and dry swab, each brushed) used on the posterior pharynx wall had a 100% concordance rate regarding positive or negative test results of SARS-CoV-2 PCR. In 9 out of 10 patients, both were positive, in 1 out of 10, both were negative. Mean Ct values of positive SARS-CoV-2 PCRs were 27.0 ±7.9 using the eSwab (Copan) with Amies transport medium, and 26.1 ±7.2 using the dry swab without transport medium (p=0.605). There was a strong correlation (R*2*=0.89, p=0.002) between the Ct values of PCR tests from the two different swab types (Figure 6 (Fig. 6)). 

In specimens taken from the posterior pharynx wall by swabs just touching the mucosa, the concordance rate was only 70%. In 5 out of 10, both were positive, in 2 out of 10, both were negative, and in 3 out of 10 there were discrepant results of SARS-CoV-2 PCRs. Mean Ct values of positive SARS-CoV-2 PCRs were 28.2 ±5.7 using eSwabs (Copan) with Amies transport medium, and 26.3 ±8.1 using dry swabs without transport medium. 

### Pharyngeal lavage

**Figure 7:**
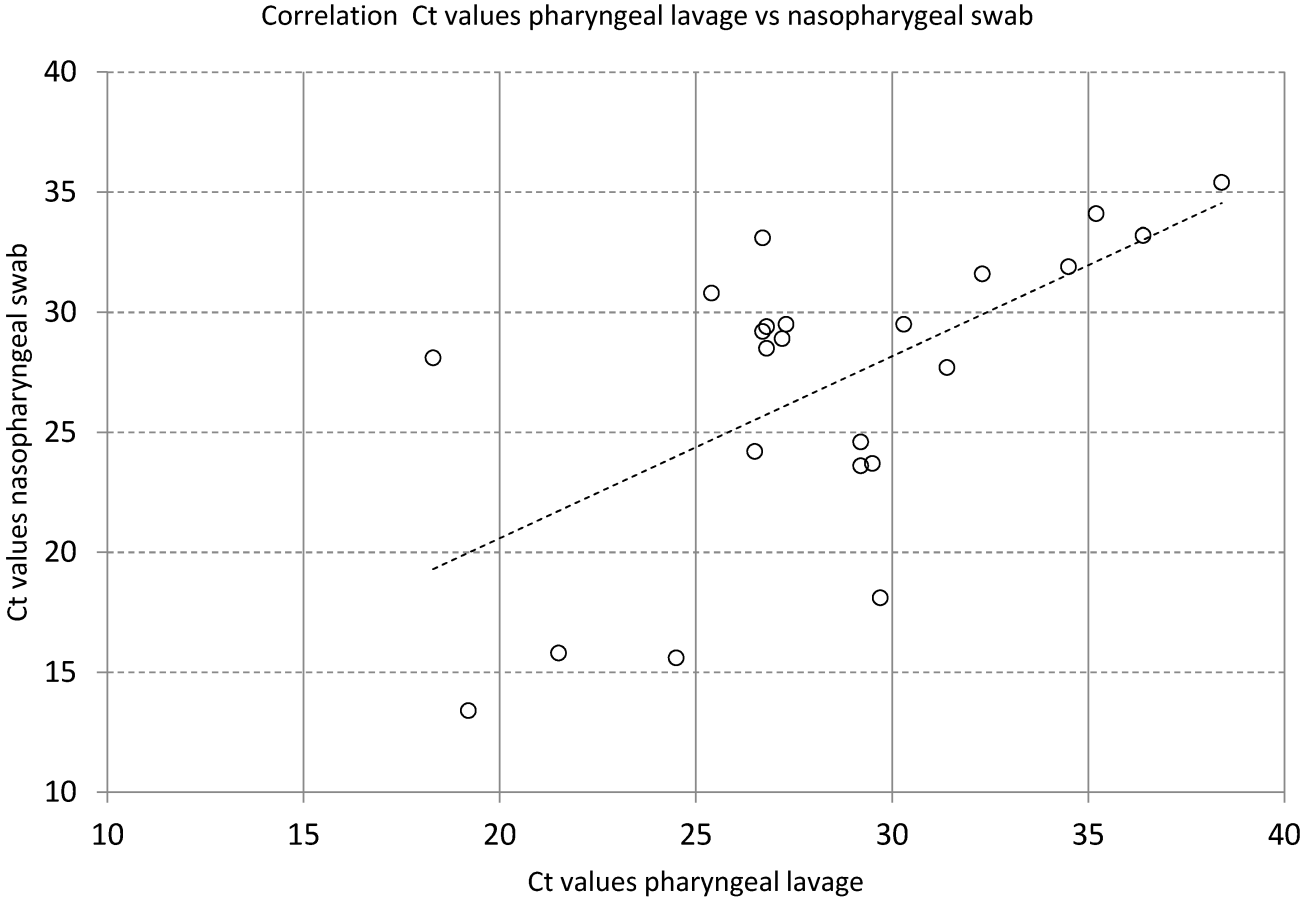
Correlation between Ct values of SARS-CoV-2 PCR from pharyngeal lavage and nasophyngeal swabs in known SARS-CoV-2-positive patients, R²=0.38

SARS-CoV-2 PCR Ct values of 23 pairs of pharyngeal lavage and nasopharyngeal swabs are given in Supplementary Table 2 . The mean Ct values of pharyngeal lavage were 28.4 ±4.91, and mean Ct values of nasopharyngeal swabs were 27.0 ±6.05. There was a strong positive correlation between these two methods of material extraction (R^2^=0.38, Figure 7 (Fig. 7)). 

### Stability of diagnostic lavage 

Pharyngeal lavage material was stored at 4°C, and SARS-CoV-2 PCR was repeated after 48 hours (Supplementary Fig. 2 ). There were no significant changes in Ct values after 48 h (25.9 ±5.0 at t0 and 26.0 ±5.3 at t48h). Variations in repeated consecutive measurements of the same lavage material are shown in Supplementary Fig. 1 . Ct values differed less than 1 Ct point from each other and were strongly correlated (R^2^=0.98). Measurements 1 and 2 had Ct values of 25.9 ±5.0 and 25.8 ±5.1, respectively.

### Vero cell viral transduction

Vero cells were transduced with SARS-CoV-2 virus particles from swabs taken of 6 patients who had severe courses of COVID-19. The SARS-CoV-2 transduced cell lines of patient 1 and 2 showed a weak cytopathogenic effect (CPE) at day 4 post inoculation (4 d.p.i.). To investigate whether SARS-CoV-2 infection was the origin of these CPEs, the supernatants of all transduced cell lines were analyzed by SARS-CoV-2 RT-PCR (Supplementary Table 3 ). Only the supernatants of patient 1 (1 d.p.i.) and patient 2 (1 d.p.i. and 6 d.p.i) showed positive RT-PCRs with high CT values compared to Ct values of the positive control. Two of the cell lines (patients 6 and 7) could not be evaluated because of fungal decay. To estimate the viral load of SARS-CoV-2 for each patient, swab material was evaluated by SARS-CoV-2 PCR prior to cell culture analysis. For all patients, high Ct values of about 30 were observed.

## Discussion

This comprehensive investigation in patients with known SARS-CoV-2 infection demonstrated new insights into the diagnostic value of different material extraction techniques and anatomical locations. Data on the duration of symptoms and length of hospital stay indicate that the tested patients were in different clinical stages of COVID-19 with declining viral load [2]. The results of SARS-CoV-2-specific antibodies support this assumption (Table 1 (Tab. 1)). However, RT-PCR results demonstrated strongly divergent Ct values. The spectrum ranged from very high (Ct <20) to very low (Ct >30) values. 

Swabs taken from the palatoglossal arch (Figure 1 (Fig. 1)) showed a diagnostic performance in detecting SARS-CoV-2 infections similar to that of the posterior pharynx region (Figure 4 (Fig. 4), both locations 90% detection rate). Both regions performed as well as or even better than nasopharyngeal swabs (85% detection rate) regarding detection of SARS-CoV-2. The main advantage of swabs taken from the palatoglossal arch is higher patient comfort (less induction of choking and coughing), and it is much easier to perform. Saliva and pharyngeal lavage yielded a lower detection rate of 50% and 60%, respectively. However, lower detection rates derive mainly from patients with high Ct values, thus assuming low viral concentrations in late infection stages. In fact, 6 out of 10 SARS-CoV-2 patients had mean Ct values of 30 or above (Figure 3 (Fig. 3)).

In the current SARS-CoV-2 pandemic, there have been supply shortages of many testing materials, including special swab kits designed for virus diagnostics (e.g. Copan eSwabs). Therefore, we tested the performance of widely available low-cost conventional dry swabs. We demonstrated that the use of dry swabs without any stabilizing transport medium is comparable to specialized swab kits (Copan eSwabs) with a modified Amies transport medium. (Figure 6 (Fig. 6), correlation coefficient between Ct-values by dry swabs versus eSwabs (Copan), R^2^=0.89). Recent data from the literature showed a comparable sensitivity for dry swabs and survival of SARS-CoV-2 on swabs stored at 4°C for at least 5 days [3]. Dry swabs have certain handling peculiarities during the PCR procedure in the medical laboratory. For RNA purification, the virus must be eluted with a liquid medium (e.g., saline solution) from the swab into a secondary sample tube. Compared to eSwabs (Copan), which can be placed directly on an RNA extraction device, the procedure is more time consuming and possibly more prone to errors (e.g., patient misidentification).

In real life, especially if swabs are taken by untrained HCW, there is a realistic probability of incorrect swabbing procedures. The unpleasant experience having a swab taken deeply from the nasopharynx or posterior pharyngeal wall will mostly lead to receding reactions. This results in abbreviated contact times of the swab with the mucosa and could subsequently result in false negative SARS-CoV-2 test results. We therefore investigated the performance of swabs just touching the posterior pharynx wall versus swab brushing the mucosa in this location. There was no difference in Ct values between posterior pharynx wall brushing technique and just touching the wall, but greater discrepancy (30%) in SARS-CoV-2 PCR results by just touching the pharyngeal wall compared to 0% discrepancy using the brushing technique at the same location. In patients with Ct values <20 using nasopharyngeal swabs, the performance of both material extraction techniques is similar. However, in patients with Ct values >30, the brushing technique with longer mucosal-contact times mucosa seems to be much more reliable, probably due to the extraction of more epithelial cells and mucosal material.

Pharyngeal lavage is an attractive alternative to nasopharyngeal or oropharyngeal swabs because it is non-invasive and low-cost, easy to self-administer and does not expose HCW to the risk of transmission, while simultaneously saving personal protective equipment. Pharyngeal lavage correlates well with nasopharyngeal swab results (Table 1 (Tab. 1), Figure 7 (Fig. 7)). It performs as well as standard swabs, but only in earlier stages of the disease (Ct values <30). The great advantage of pharyngeal lavage is its simplicity of collection and the easy and inexpensive availability of the materials needed. Even screening of large cohorts of persons can be performed without challenging logistics, by providing a receptacle and 10 mL saline solution to each person to be tested and collecting closed receptacles containing the gargled fluid, even outside of medical facilities (e.g., retirement homes). Drinking or eating did not negatively influence the detection rate of pharyngeal lavage. Repetition of SARS-CoV-2 PCR from pharyngeal lavage after 48 h storage at 4°C proved that diagnostic accuracy is maintained for at least 2 days (Supplementary Fig. 2 ). We additionally demonstrated the extremely low variability in Ct values from the same material (Supplementary Fig. 1 ). In patients with Ct value >30, pharyngeal lavage performed significantly worse regarding SARS-CoV-2 positivity compared to nasopharyngeal swabs or swabs taken from the palatoglossal arch (Figure 4 (Fig. 4)). 

Swabs from the buccal mucosa and saliva had the lowest probability of correctly detecting SARS-CoV-2 positivity. The low performance of saliva contrasts with recent data demonstrating similar performance [4], [5] or even higher sensitivity [6] compared to nasopharyngeal swabs. Different stages of infection within patient cohorts could be one explanation for the contradicting results. 

The cell culture analysis showed no correlation between CPE and RT-PCR positivity. This indicates that the observed CPEs are not caused by SARS-CoV-2 infection of the Vero cells. The BetaCoV/Germany/BavPat1/2020 transduced positive-control strain showed a strong correlation of 100% with clear cut CPEs and very low Ct-Values of SARS-CoV2-PCR. The positive RT-PCR results with high Ct-Values in 3 of the supernatants may be due to remaining inactive virus from the original patient swab. In agreement with recent reports, the results showed that specimens of patients with low SARS-CoV-2 concentrations (Ct >30) are unable to infect cell cultures [2]. 

## Notes

### Competing interests

The authors declare that they have no competing interests.

### Acknowledgements

We would like to thank the brave nurses of the Clinic for Infectiology in our hospital for their untiring commitment during the difficult pandemic times.

### Funding

The authors did not receive any external funding

### Authorship

Ralph Wendt and Olaf Nickel have shared first authorship.

## Supplementary Material

Supplementary

## Figures and Tables

**Table 1:**
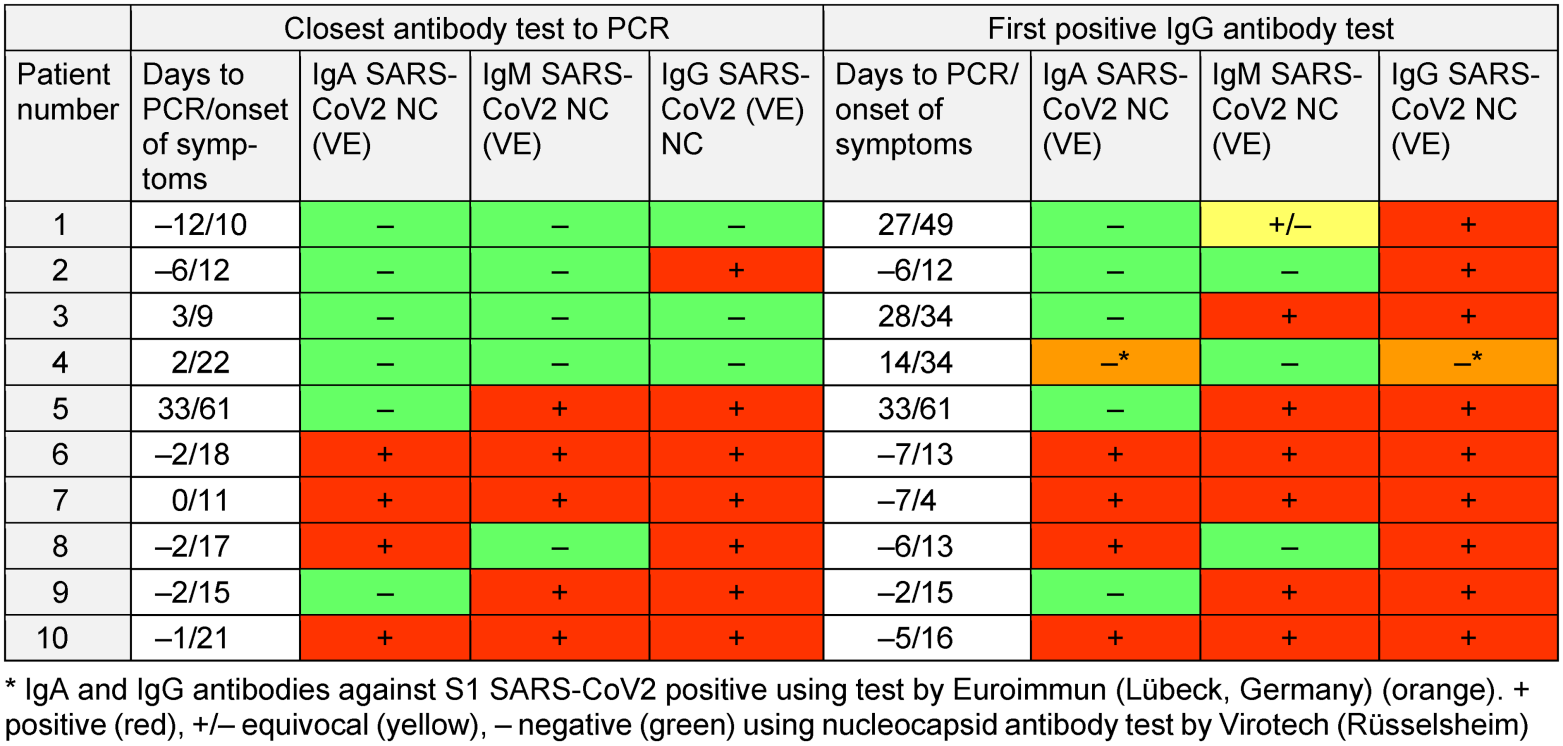
Antibody results
