# Supplementary material for: Comparison of pre-analytical characteristics for molecular and serological diagnostics of COVID-19
Source: GMS Hyg Infect Control. 2021 Jan 19;16:Doc03. doi: 10.3205/dgkh000374 (PMC7818659; doi:10.3205/dgkh000374)
Supplement: Supplementary [file HIC-16-03-s-001.pdf]

Supplementary

Ct pharyngeal lavage	Ct nasophyryngeal swab
18,3	28,1
29,5	23,7
26,5	24,2
32,3	31,6
34,5	31,9
31,4	27,7
26,7	29,2
27,3	29,5
26,8	28,5
29,2	23,6
27,2	28,9
30,3	29,5
25,4	30,8
29,2	24,6
26,8	29,4
26,7	33,1
36,4	33,2
21,5	15,8
19,2	13,4
38,4	35,4
29,7	18,1
24,5	15,6
35,2	34,1

Supplementary Table 2: 23 pairs of corresponding Ct values of pharyngeal lavage and nasopharyngeal swabs

	1 d.p.i		3 d.p.i		P1 4 d.p.i		P2 6 d.p.i.	Patient Sample	
	Ct	CPE	Ct	CPE	Mean Ct	CPE	Mean Ct	CPE	Ct
**Patient 1**	34.2	?	n.a.	–	n.a.	(+)	n.a.	–	27,5
**Patient 2**	31.8	?	n.a.	–	n.a.	(+)	33.7	–	32.2
**Patient 3**	n.a.	?	n.a.	–	n.a.	–	n.a.	–	31.8
**Patient 4**	n.a.	?	n.a.	–	n.a.	–	n.a.	–	29.8
**Patient 5**	n.a.	?	n.a.	? (F)	n.a.	? (F)	n.a.	? (F)	37.2
**Patient 6**	n.a.	?	n.a.	? (F)	n.a.	? (F)	n.a.	? (F)	35.5
**pos Ctrl**	11.8	?	10.4	–	9.4	+	6.2	+++	
**neg Ctrl**	n.a.	?	n.a.	–	n.a.	–	n.a.	–	

Supplementary Table 3: Comparison between Ct–Values of SARS–CoV–2 PCR and cytopathic effects (CPE) in SARS–CoV–2–inoculated Vero E6 cells at days 1–6 post inoculation (d.p.i.). Cells were inoculated with throat swab material of 6 SARS–CoV–2 positive patients. The samples of 2 patients could not be evaluated because of fungal decay (F). Samples after the passage 1 and 2 (P1 and 2) were analyzed in duplicates. The results are given as mean values. To estimate the viral load of SARS–CoV–2 for each patient, swab material was evaluated by SARS–CoV–2 PCR prior to the cell culture analysis.

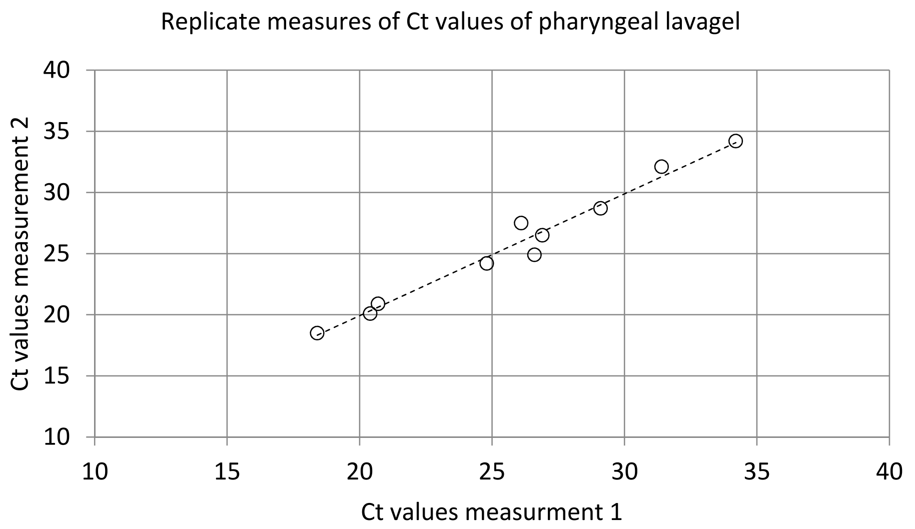

Supplementary Fig. 1: Variability of Ct values of repeated SARS–CoV–2 PCR from pharyngeal lavage, R^2=0.97

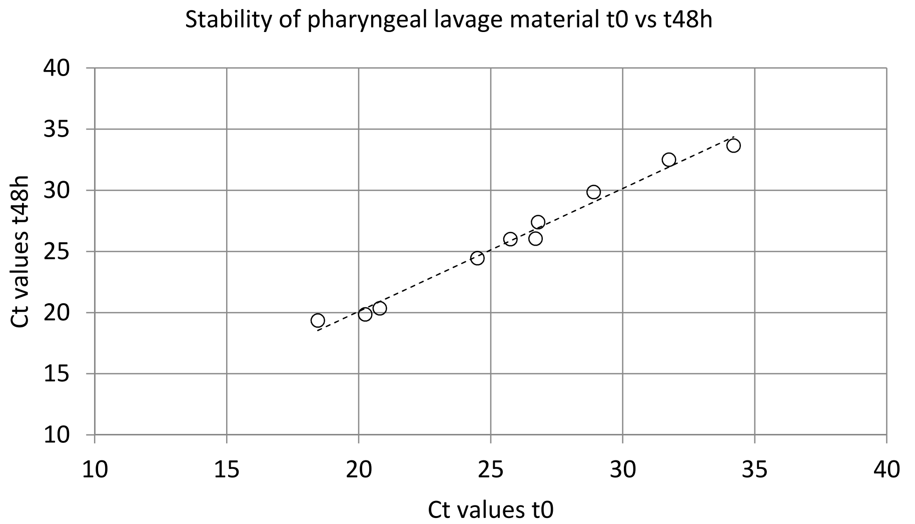

Supplementary Fig. 2: Variability of Ct values of repeated SARS–CoV–2 PCR over time from pharyngeal lavage at baseline and after 48h (stored at 4°C), R^2=0.98

Attachment 1 to: Wendt R, Nickel O, Kalbitz S, Fertey J, Ulbert S, Wolf J, Lübbert C, Borte S. Comparison of pre-analytical characteristics for molecular and serological diagnostics of COVID-19. GMS Hyg Infect Control. 2021;16:Doc03. DOI: 10.3205/dgkh000374
